# The relationship between college students’ perceived physical education environment and student engagement: a latent profile and structural equation modeling analysis

**DOI:** 10.3389/fspor.2025.1698164

**Published:** 2026-01-07

**Authors:** Anlin Guan, Liguo Zhang, Huizhuo Wu

**Affiliations:** 1 School of Physical Education and Health Sciences, Guangxi Minzu University, Nanning, China; 2 School of Physical Education, Shandong University, Jinan, China

**Keywords:** academic self-efficacy, perceived physical education environment, perceived teacher support, physical education, student engagement

## Abstract

**Background:**

College students’ participation in physical education (PE) classes is of great significance to teaching effectiveness and students’ physical and mental health. However, few studies have explored the influencing factors and mechanisms of PE class engagement. This study aims to investigate the association between college students’ perceived PE environment and their PE class engagement, as well as the mediating roles of perceived teacher support and academic self-efficacy.

**Methods:**

A cross-sectional survey was conducted among 1,158 Chinese college students from December 1 to 31, 2024. Variables were measured using the Perceived Physical Education Environment Scale, Perceived Teacher Support Scale, Academic Self-Efficacy Scale, and Classroom Engagement Scale. Mplus 8.11 was used to perform latent profile analysis on the perceived PE environment, which classified the participants into three groups: low-perception group, medium-perception group, and high-perception group. Amos 28.0 was employed to construct a structural equation model, and SPSS 26.0 was used to test the mediating effects.

**Results:**

In the total sample and the three groups, the perceived PE environment was significantly and positively correlated with college students’ PE class engagement (*p* < 0.01). In the medium-perception and high-perception groups of PE environment, perceived teacher support and academic self-efficacy played both individual mediating roles and a chain mediating role in the relationship between perceived PE environment and PE class engagement. However, in the low-perception group of PE environment, the mediating role of perceived teacher support was not significant, while the chain mediating role remained significant.

**Conclusion:**

This study identifies that perceived teacher support and academic self-efficacy are key mechanisms linking the perceived PE environment to students’ class engagement, and this sequential mechanism is particularly important in the context of low perceived PE environment. The results of this study emphasize the practical significance of strengthening school PE environments and resources, improving teachers’ guidance and support, and fostering students’ academic self-efficacy for enhancing students’ class engagement.

## 1 Introduction

With societal development and the growing pursuit of healthier lifestyles, physical education (PE) has become increasingly prominent in higher education. As the primary vehicle for university PE, PE classes depend critically on student engagement, which is closely linked to instructional effectiveness and the cultivation of students' physical literacy (1). However, student engagement in PE classes among Chinese undergraduates remains generally low, a situation that may undermines students' physical health and constrains the development of university PE (2). Identifying factors associated with PE class engagement therefore has substantial theoretical and practical significance. Student engagement in PE classes—an important indicator of instructional quality and a key correlate of students' physical and mental health—is shaped by multiple influences, among which students’ perceptions of the PE environment are particularly salient. The perceived PE environment refers to students' subjective appraisals of curriculum design, teacher guidance, material (physical) conditions, and institutional arrangements within the university setting (3). More favorable perceptions are associated with more active participation in physical activity, richer social interaction, and better learning outcomes (4). Prior research further indicates that perceived teacher support and academic self-efficacy are important correlates of student engagement (5, 6), and these factors also appear to be relevant in PE contexts. Although existing studies have examined the association between the perceived PE environment and student engagement, most have been limited to direct-effect analyses and have not explored the underlying mechanisms in depth (7). In the context of Chinese higher education, cultural and institutional features may impart distinctive characteristics to these mechanisms. For example, the authoritative role of teachers and a collectivist orientation may amplify students' perceived teacher support, whereas intense academic competition may heighten the relevance of academic self-efficacy for PE participation. Against this backdrop, the present cross-sectional study examines the relationships among the perceived PE environment, perceived teacher support, academic self-efficacy, and undergraduates' engagement in PE classes, with the aim of providing theoretical grounding and practical guidance for the construction of university PE environments and for instructional reform. In doing so, the study applies ecological systems theory, social support theory, and social cognitive theory to the field of PE, and offers a potential intervention-oriented perspective for addressing low engagement in university PE.

### 1.1 Influence of undergraduates' perceived physical education environment on student engagement

Perceived PE Environment refers to students' subjective evaluations formed through interactions with the various elements of the PE context. It encompasses four dimensions: curricular structure (e.g., intensity, interest, and appropriateness of PE courses), teacher guidance (e.g., instructional approaches, responsive feedback, and provision of opportunities), institutional environment (e.g., school-provided PE guidance and training services), and material environment (e.g., availability of sports venues and equipment) (3). According to ecological systems theory, human behavior and psychological development are closely linked to the environment: individual behavior is shaped by the environment and, in turn, shapes it (8). Empirical research indicates that undergraduates' perceived PE environment are positively associated with their participation in physical activity (7) and, through such participation, further influence their gains from PE (9). Student engagement is generally defined as the time and effort students invest in learning at school and includes behavioral, emotional, and cognitive components. Behavioral engagement refers to students' participation in academic or school-based extracurricular activities and is related to their on-task behavior in class; emotional engagement includes students' positive and negative feelings toward school, learning, teachers, and classmates, and is associated with their willingness and attitudes toward learning; cognitive engagement refers to students' willingness to exert effort to learn and understand knowledge and is typically related to self-efficacy and achievement goals (10). Engagement in PE classes refers to the time and effort students devote to PE learning and activities in the school setting. It places greater emphasis on practical activities and highlights students' active participation in physical exercise, concentration, interest, and the learning and mastery of motor skills. In this study, cognitive engagement in PE refers to students' concentration and persistence when participating in physical activities, as well as their tendency to attempt to master challenging motor skills. Behavioral engagement refers to students' active participation and positive behaviors in PE classes; and emotional engagement refers to students' interest in and positive emotions toward participating in physical activities during PE lessons (9). A substantial body of work on PE classroom environments indicates that such environments are associated with students' interest in participation, their perceived competence, and teacher–student interactions, with consequences for behavioral, cognitive, and emotional outcomes (11). Curricular structure is regarded as a key educational–environmental factor in school PE and is closely related to the cultivation of students' interest and the effectiveness of instruction and training (12). In addition, studies have found that teacher-guided instructional settings are associated with stronger collaborative problem-solving abilities among students (13). Sports equipment and facilities, as elements of the material environment, are widely considered critical in school PE: the adequacy and quality of facilities are closely related to instructional effectiveness and students' enthusiasm for autonomous exercise outside of class (14). One study further suggests that policies supporting the PE environment are associated with more active engagement in physical activity (15). Moreover, some scholars argue that, compared with objective environmental features, subjective perceptions of the school PE environment may more strongly motivate individuals' willingness to participate (16). Therefore, undergraduates' perceived PE environment are likely to be associated with higher levels of engagement in PE classes.

### 1.2 Mediating role of perceived teacher support between perceived physical education environment and student engagement

Perceived teacher support refers to students' subjective cognitions and affective experiences regarding teachers' attitudes and behaviors toward their learning and daily lives, encompassing learning support, emotional support, and competence support (17). Learning support denotes the learning-related assistance that teachers provide inside and outside the classroom; emotional support refers to the respect, care, and acceptance conveyed by teachers in their interactions with students; and competence support describes the ways in which teachers use appropriate expectations and guidance to help students develop a sense of competence and confidence. According to social support theory, individuals' social relationships and support networks are crucial for well-being; receiving emotional support, informational support, tangible assistance, and supportive relationships has been associated with better physical and mental health (18). Empirical studies have documented significant associations between perceived teacher support and student engagement. A review in the context of PE reports a positive correlation between perceived teacher support and student participation in PE (5). Students who report higher levels of teacher support typically exhibit greater interest and initiative in class and higher engagement, which, in turn, are linked to better academic performance, whereas lower perceived support may be associated with reduced engagement (19). Research further suggests that course goals within curricular design are related to the nature of teacher support, as teachers are more likely to design targeted supportive behaviors around clearly defined objectives (20). The greater the perceived alignment between course goals and students' personal needs, the more favorable their evaluations of teacher support. A cross-school study reports that schools implementing quantitative evaluation systems for teacher behavior tend to show higher levels of perceived teacher support (21). In addition, greater financial investment in PE appears to provide teachers with conditions that facilitate supportive activities, thereby enhancing students' perceptions of teacher support (22, 23). Because the perceived PE environment encompass curricular structure, teacher guidance, institutional arrangements, and material conditions, such perceptions may be related to the extent of support that teachers provide and, in turn, to students' perceptions of teacher support.

### 1.3 Mediating role of academic self-efficacy between perceived physical education environment and student engagement

Academic self-efficacy refers to individuals' beliefs that they can effectively deploy self-regulatory strategies to accomplish educational goals, encompassing confidence in both learning abilities and behaviors (24). In the present study, academic self-efficacy denotes individuals' belief that they can effectively use self-regulatory strategies to meet the educational goals of PE courses, including confidence in PE-related learning abilities as well as in their performance of physical behaviors. According to social cognitive theory, individuals with high self-efficacy tend to be confident, willing to confront difficulties and challenges, and persistent in pursuing their goals; they display greater interest, perseverance, and investment in activities and are therefore more likely to achieve favorable outcomes, which may, in turn, further strengthen self-efficacy (25). Empirical evidence indicates that academic self-efficacy is positively associated with student engagement (26). Students with higher academic self-efficacy have been found to show greater enthusiasm and investment in classroom learning and to attain better academic performance (27), which may in turn be associated with increases in self-efficacy. Although academic self-efficacy has most commonly been examined in relation to core academic subjects such as mathematics or language arts, social cognitive theory holds that self-efficacy is, in essence, a judgment of confidence with respect to a specific class of tasks or situations. Extending this construct to the PE classroom context and focusing on students' confidence in mastering PE-related knowledge, skills, and course requirements is therefore fully consistent with social cognitive theory. Perceived PE environment refer to students' views and psychological appraisals of that environment. Scholars have found that undergraduates’ perceptions of the classroom environment are significantly associated with their self-efficacy and satisfaction. When students perceive alignment between course objectives and their own ability levels, they have been more likely to complete learning tasks and gain successful experiences (28), which may contribute to higher levels of academic self-efficacy. In addition, teachers' feedback, guidance, and encouragement have been linked to reduced exercise-related anxiety, stronger beliefs about motor competence, and higher self-efficacy (29). The material conditions of school PE provide resources and safeguards; research indicates that adequate, high-quality, and safe sports facilities are associated with lower emotional arousal and stronger efficacy beliefs (30). Therefore, perceived PE environment may be related to students' academic self-efficacy and, in turn, to their engagement in PE classes.

### 1.4 Chain mediating roles of perceived teacher support and academic self-efficacy between perceived physical education environment and student engagement

Social cognitive theory posits that human behavior results from reciprocal interactions among the environment, the individual, and behavior (25), and that self-efficacy is shaped by multiple contextual information sources, such as mastery experiences, vicarious experiences, and social persuasion. Undergraduates' engagement in PE classes is closely linked to the external environment, including the on-campus PE context and teacher support. A substantial body of research reports significant associations between perceived teacher support and academic self-efficacy (31). Through positive feedback, clear expectations, timely guidance, and emotional encouragement, teachers may foster higher levels of perceived teacher support, which are in turn associated with greater confidence in academic abilities and higher academic self-efficacy (32). A study conducted in China found that academic support provided by teachers was associated with higher perceptions of students' own academic abilities, particularly when students faced academically challenging tasks (33). Another study reported that, in positive and highly supportive sports environments, students tended to report higher levels of self-efficacy in motor learning and performance (34). Academic self-efficacy has been identified as an important determinant-like correlate of students' interest and engagement and is strongly linked to learning engagement and academic achievement. Several studies suggest that perceived teacher support is related to student engagement in part through the mediating role of academic self-efficacy (9, 35). In the field of PE, recent research has further indicated that teacher support is associated with higher PE learning engagement via academic self-efficacy (36). Accordingly, perceived teacher support is often regarded as a socio-interpersonal manifestation of the PE environment that precedes and shapes students' academic self-efficacy at the conceptual level. Students first encounter teachers' expectations, feedback, and encouragement in social interactions and then internalize this information as beliefs about their ability to succeed in PE-related learning tasks, which may promote greater effort, persistence, and emotional engagement in PE classes. Because perceived PE environment encompass curricular structure, teacher guidance, institutional context, and material conditions, such perceptions may be related to the extent of support that teachers provide and, in turn, to students' perceptions of teacher support. On this basis, students' perceived PE environment are expected to be associated with classroom engagement via perceived teacher support and academic self-efficacy, rather than implying strictly causal effects.

**Figure 1:**
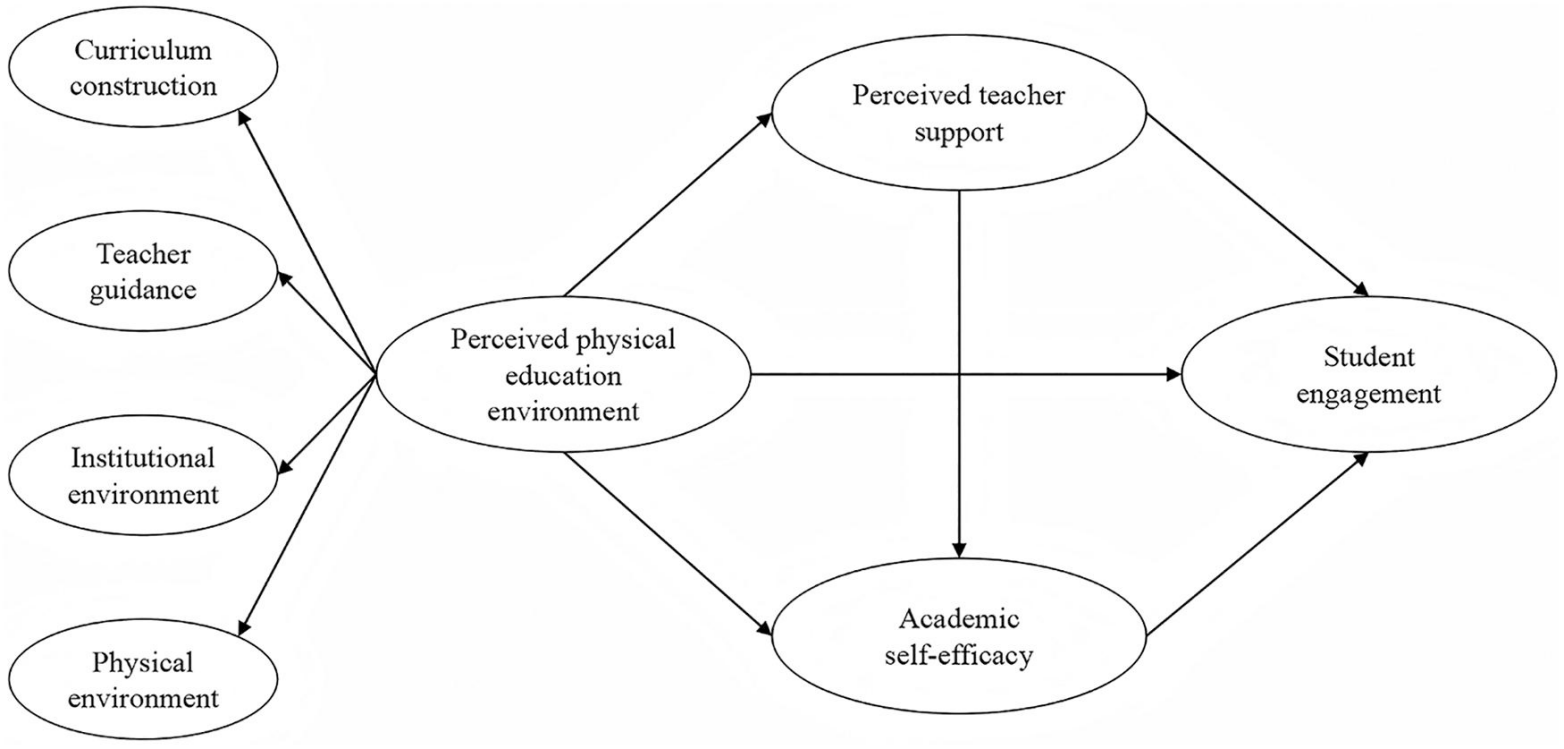
Hypothetical model diagram.

Focusing on Chinese undergraduates and drawing on ecological systems theory, social support theory, and social cognitive theory, this cross-sectional study examines associations between differing levels of the perceived PE environment and engagement in PE classes, as well as the potential mediating roles of perceived teacher support and academic self-efficacy in these associations. Accordingly, we formulated the following hypotheses (Figure 1):

Hypothesis 1: Undergraduates’ perceived PE environment are positively associated with student engagement in PE classes.
Hypothesis 2: Perceived teacher support mediates the association between the perceived PE environment and student engagement.
Hypothesis 3: Academic self-efficacy mediates the association between the perceived PE environment and student engagement.
Hypothesis 4: Perceived teacher support and academic self-efficacy play a chain mediating role in the association between the perceived PE environment and student engagement.

## 2 Method

### 2.1 Participants and procedure

Using convenience and snowball sampling, we conducted a cross-sectional survey of university students across China between 1 and 31 December 2024. In total, 1,612 questionnaires were collected. After excluding cases with missing data and submissions with completion times <15 min, 1,158 valid questionnaires remained. The survey was administered via the online platform “Wenjuanxing” (https://www.wjx.cn/). Prior to participation, all respondents were informed of the purpose of the study; anonymity and confidentiality were assured, results were used solely for research purposes, and informed consent was obtained. The study complied with the Declaration of Helsinki and was approved by the Ethics Committee of the School of Basic Medical Sciences, Shandong University (approval number ECSBMSSDU2022-1-086). Of the included participants, 717 (61.92%) were male and 441 (38.08%) were female. A total of 37 students (3.20%) were younger than 18 years, 1,115 (96.29%) were aged 18–25 years, and 6 (0.52%) were older than 25 years. There were 653 first-year undergraduates (56.39%), 402 s-year students (34.72%), 91 third-year students (7.86%), and 12 fourth-year students (1.04%).

### 2.2 Measures

#### 2.2.1 Perceived physical education environment

The perceived PE environment were assessed using the School Environment Perception Scale (3), which was developed on the basis of student engagement theory and revised with reference to the National Survey of Student Engagement (NSSE), the College Student Experiences Questionnaire (CSEQ), and NSSE-China. The scale comprises four dimensions—curricular structure (e.g., “PE courses stimulate your interest in learning”), teacher guidance (e.g., “In PE classes, teachers use heuristic and interactive teaching methods”), institutional environment (e.g., “Your university has effective channels for expressing students’ views and provides timely feedback”), and material environment (e.g., “Your university has abundant and easily accessible sports facilities”)—with a total of 19 items. Items were rated on a 5-point Likert scale from 1 (“strongly disagree”) to 5 (“strongly agree”); item scores were summed to yield a total score, with higher scores indicating more favorable perceptions. Cronbach's α was 0.972.

#### 2.2.2 Perceived teacher support

Perceived teacher support was measured using the questionnaire developed by Ouyang (37). This instrument includes three dimensions: emotional support (e.g., “My teachers often encourage me in my studies and daily life”), learning support (e.g., “In my studies, my teachers place high demands on me”), and competence support (e.g., “My teachers believe that I am always capable of completing difficult assignments or tasks”), with 19 items in total. Each item was rated on a 5-point Likert scale ranging from 1 (“strongly disagree”) to 5 (“strongly agree”). Higher total scores reflect higher levels of perceived teacher support. Cronbach's α was 0.939.

#### 2.2.3 Academic self-efficacy

Academic self-efficacy was assessed using a scale based on Pintrich and DeGroot (38) and adapted and revised by Liang (39). The scale comprises two dimensions: self-efficacy for learning behaviors (e.g., “When studying, I like to ask myself questions to check whether I have mastered the material”) and self-efficacy for learning ability (e.g., “I believe I am capable of achieving good results in my studies”), with 11 items in each dimension and 22 items in total. For the purposes of the present study, the original items were further adapted to the PE classroom context so that the scale captured students' domain-specific academic confidence in relation to PE courses, learning tasks, and grades, rather than global academic self-efficacy across all subjects. All items were rated on a 5-point Likert scale from 1 (“strongly disagree”) to 5 (“strongly agree”). Total scores were calculated by summing all items, with higher scores indicating higher academic self-efficacy. Cronbach's α was 0.927.

#### 2.2.4 Student engagement

Student engagement in PE classes was evaluated using the Student Classroom Engagement Scale, which covers cognitive engagement (e.g., “In PE classes, I listen carefully”), behavioral engagement (e.g., “When doing assignments in PE classes, I try to connect what I am learning with what I already know”), and emotional engagement (e.g., “I am interested in the content being taught”) (40). The instrument comprises 10 items rated on a 5-point Likert scale from 1 (“strongly disagree”) to 5 (“strongly agree”); item scores were summed to produce a total score, with higher scores indicating higher student classroom engagement. Cronbach's α was 0.972.

### 2.3 Statistical analysis

Data were analyzed using SPSS 26.0 (IBM Corporation, Armonk, NY, USA), Mplus 8.11 (Muthén & Muthén, Los Angeles, CA, USA), and Amos 28.0 (IBM Corporation, Armonk, NY, USA). Categorical variables were summarized as counts and percentages, and continuous variables were summarized as means ± standard deviations. Harman's single-factor test was first applied to assess common method bias, followed by descriptive statistics and Pearson correlation analyses in SPSS. Latent profile analysis was conducted in Mplus to group participants according to their perceived PE environment. After the optimal profile model had been identified, individual posterior probabilities were used to assign participants to different latent profiles, and this categorical classification was then used in subsequent analyses, thereby linking the person-centered LPA results with the variable-centered structural equation models. Model selection was based on the Akaike information criterion (AIC), Bayesian information criterion (BIC), sample size–adjusted BIC (aBIC), entropy, the bootstrap likelihood ratio test (BLRT), and the Lo–Mendell–Rubin (LMR) test. Smaller AIC, BIC, and aBIC values indicated better fit; entropy >0.80 indicated satisfactory classification quality; and *p* < 0.05 in the BLRT and LMR tests suggested that the k-class model was superior to the k−1 class model (41). Structural equation modeling was performed in Amos, and model fit was evaluated using the chi-square to degrees-of-freedom ratio (*χ*^2^/df), root mean square error of approximation (RMSEA), goodness-of-fit index (GFI), adjusted goodness-of-fit index (AGFI), normed fit index (NFI), incremental fit index (IFI), comparative fit index (CFI), and Tucker–Lewis index (TLI). Finally, mediation and serial mediation effects of perceived teacher support and academic self-efficacy in the association between the perceived PE environment and student classroom engagement were tested in SPSS using PROCESS macro 4.3 (Models 4 and 6), with bias-corrected bootstrapping (*n* = 5,000). Mediation was considered significant if the 95% confidence interval did not include zero.

## 3 Results

### 3.1 Common method bias test

Because all questionnaires were self-reported, potential common method bias was assessed using Harman's single-factor test across the four validated instruments (47 items). Eight factors had eigenvalues greater than 1.0, and the variance explained by the first factor was 34.096%, below the 40% threshold. These results suggest that serious common method bias was unlikely.

### 3.2 Descriptive and correlation analyses

Table 1 presents descriptive statistics for all variables. The sample comprised predominantly men (717, 61.92%), students aged 18–25 years (1,115, 96.29%), individuals of Han ethnicity (1,043, 90.07%), first-year undergraduates (653, 56.39%), and residents of East China (393, 33.94%). Table 2 reports Pearson correlations among perceived PE environment, perceived teacher support, academic self-efficacy, and student engagement; all pairwise associations were significantly positive (*p* < 0.01), providing the statistical basis for subsequent analyses.

**Table 1:** Descriptive analysis.

Variables	Options	Number %/Mean ± SD	
Sex	Female	441	38.08
	Male	717	61.92
Age	≤18 years old	37	3.20
	18∼25 years old	1,115	96.29
	26∼30 years old	4	0.35
	≥30 years old	2	0.17
Nation	Han nationality	1,043	90.07
	Minority	115	9.93
Grade	First-year undergraduate	653	56.39
	Second-year undergraduate	402	34.72
	Third-year undergraduate	91	7.86
	Fourth-year undergraduate	12	1.04
Address	East China	393	33.94
	South China	183	15.80
	Central China	329	28.41
	North China	10	0.86
	Northwest China	1	0.09
	Southwest China	84	7.25
	Northeast China	158	13.64
Student engagement		41.586 ± 7.139	
Perceived physical education environment	Course construction	20.895 ± 3.304	
	Teacher guidance	20.870 ± 3.352	
	Institutional environment	12.536 ± 2.046	
	Physical environment	16.349 ± 2.986	
Perceived teacher support	Learning support	34.545 ± 5.108	
	Emotional support	24.167 ± 3.810	
	Capacity support	10.294 ± 2.260	
Academic self-efficiency	Learning ability self-efficacy	43.699 ± 7.614	
	Learn about self-efficacy in learning	40.168 ± 6.116	

**Table 2:** Pearson correlation analysis.

Variables	Mean	SD	1	2	3	4
1. Perceived physical education environment	70.649	10.767	–			
2. Perceived teacher support	69.006	10.390	0.764**	–		
3. Academic self-efficacy	83.867	12.400	0.734**	0.695**	–	
4. Student engagement	41.586	7.139	0.716**	0.662**	0.628**	–

** *p* < 0.01.

### 3.3 Latent profile analysis

**Figure 2:**
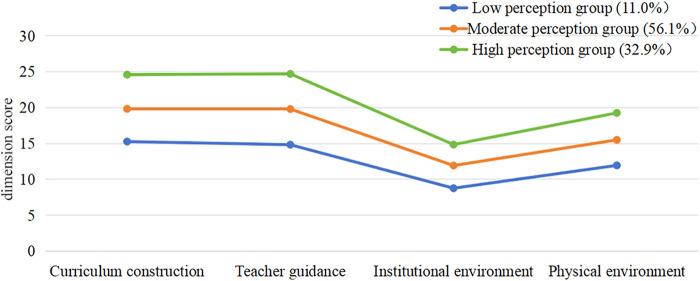
Dimension score trajectories by latent classes of perceived PE environment.

Latent profile analysis was conducted to classify participants according to their levels of perceived PE environment. Model fit indices are shown in Table 3. The two-, three-, four-, and five-profile solutions all showed high classification quality, with entropy values exceeding 0.80. As the number of profiles increased, AIC, BIC, and aBIC decreased. For the two-, three-, and four-profile solutions, the LMR and BLRT tests yielded *p* < 0.05; however, for the five-profile solution, the LMR p-value was 0.458 (>0.05) and thus not significant. In addition, in the four-profile solution, the smallest class contained only six individuals, precluding reliable statistical analysis. Therefore, after comprehensive comparison, the three-profile model was selected as the optimal solution. On the basis of this three-profile model, participants were categorized into low-, moderate-, and high-perception groups, with sample sizes of 125 (10.8%), 652 (56.3%), and 381 (32.9%), respectively (Fig. 2). Descriptive statistics and analysis of variance for the key variables across the three groups are presented in Table 4. The results indicated significant group differences in perceived PE environment, perceived teacher support, academic self-efficacy, and student classroom engagement, suggesting that the LPA-derived profiles reflected substantive heterogeneity rather than mere statistical artifacts. Accordingly, in the subsequent structural equation modeling and mediation analyses, the low-, moderate-, and high-perception profiles were treated as conceptually distinct subgroups to examine whether the associations among the key variables varied as a function of profile membership.

**Table 3:** Model fit indices for the latent profile analysis.

Number of profiles	AIC	BIC	aBIC	LMR (*p*)	BLRT (*p*)	Entropy	Minimum class size (Proportion)
2	20,168.975	20,234.683	20,193.390	0.000	0.000	0.971	392 (33.9%)
3	17,869.569	17,960.549	17,903.375	0.004	0.000	0.982	127 (11.0%)
4	17,356.365	17,472.617	17,399.562	0.035	0.000	0.987	6 (0.5%)
5	17,084.793	17,226.317	17,137.380	0.458	0.000	0.987	6 (0.5%)

AIC, Akaike information criterion; BIC, Bayesian information criterion; aBIC, adjusted Bayesian information criterion; LMR, Lo-Mendell-Rubin; BLRT, bootstrap likelihood ratio test.

**Table 4:** Descriptive analysis of key variables after division into three groups.

	Low perception group		Moderate perception group		High perception group		
Variables	Mean	SD	Mean	SD	Mean	SD	*p*
PPEE	50.62	5.122	67.03	3.200	83.41	3.011	0.000
PTS	55.16	7.406	66.00	6.234	78.69	8.472	0.000
ASE	67.52	8.830	80.60	8.015	94.82	10.302	0.000
SE	33.18	8.254	39.54	4.844	47.85	4.797	0.000

### 3.4 Structural equation modeling

Using Amos, we specified a structural equation model with perceived PE environment as the independent variable, student classroom engagement as the dependent variable, and perceived teacher support and academic self-efficacy as mediators. Sex, academic year, place of residence, and major were included as control variables. The model was estimated in the total sample and separately in the low-, moderate-, and high-perception groups derived from the LPA. Model fit indices are reported in Table 5, and most indices fell within acceptable ranges, indicating satisfactory overall fit. Path analyses showed a significant positive association between perceived PE environment and student classroom engagement (*B* = 0.476, *p* < 0.01), consistent with Hypothesis 1. In addition, perceived PE environment was positively associated with perceived teacher support (*B* = 0.731, *p* < 0.01) and academic self-efficacy (*B* = 0.559, *p* < 0.01). Perceived teacher support and academic self-efficacy were each positively associated with student engagement (*B* = 0.162, *p* < 0.01; *B* = 0.091, *p* < 0.01).

**Table 5:** Structural equation model fitting index.

Fit indices	All participants	Low perception group	Moderate perception group	High perception group	Reference ranges
x^2^/df	3.288	1.088	1.999	1.324	<3
RMSEA	0.044	0.027	0.039	0.029	<0.06
GFI	0.987	0.962	0.986	0.985	>0.90
AGFI	0.974	0.925	0.972	0.969	>0.90
NFI	0.981	0.845	0.903	0.921	>0.90
IFI	0.986	0.985	0.949	0.979	>0.90
CFI	0.986	0.984	0.948	0.979	>0.90
TLI	0.979	0.975	0.919	0.967	>0.90

### 3.5 Mediation analysis

Building on the latent profile groupings and the satisfactory SEM fit, we used PROCESS (Models 4 and 6) in SPSS to examine mediation and serial mediation for the full sample and for the low-, moderate-, and high-perception groups. As shown in Table 6, the total association between perceived PE environment and student engagement was significantly positive in all groups (*p* < 0.001), with the largest estimate observed in the high-perception group. In the full sample, both perceived teacher support and academic self-efficacy showed significant indirect and serial indirect associations between perceived PE environment and student engagement [95% CI = (0.020, 0.058), *p* = 0.010]. This pattern is consistent with Hypotheses 3, 4, and 5. In the low-perception group, the direct association between perceived PE environment and student engagement was not significant, and the indirect pathway through perceived teacher support alone was also not significant; however, the serial pathway via perceived teacher support and academic self-efficacy was significant [95% CI = (0.009, 0.121), *p* = 0.018], consistent with a pattern of full mediation in this subgroup. In the moderate- and high-perception groups, perceived teacher support and academic self-efficacy each showed significant indirect and serial indirect paths [95% CI = [0.001, 0.020], *p* = 0.003; 95% CI = [0.007, 0.046], *p* < 0.001], consistent with partial mediation in both groups. Thus, in the low-perception profile, perceived PE environment appears to be related to student classroom engagement primarily through the joint pathways involving perceived teacher support and academic self-efficacy, whereas in the moderate- and high-perception profiles, perceived teacher support and academic self-efficacy function in parallel as well as jointly. Integrating LPA with mediation analysis therefore provides a more fine-grained delineation of the statistical pathways through which perceived PE environment is associated with students' classroom engagement in this cross-sectional sample.

**Table 6:** Chain mediating effect test.

Groups	Paths	Effect	Boot SE	BootLLCI	BootULCI	*z*/*t*	*p*	Contribution
All participants	PPEE→SE (direct effect)	0.282	0.022	0.238	0.326	12.654	0.000	–
	PPEE→PTS→SE	0.118	0.028	0.127	0.235	4.173	0.000	60.8%
	PPEE→ASE→SE	0.051	0.018	0.041	0.116	2.791	0.005	26.3%
	PPEE→PTS→ASE→SE	0.025	0.010	0.020	0.058	2.562	0.010	12.9%
	PPEE→SE (total effect)	0.476	0.014	0.45	0.503	35.007	0.000	–
Low perception group	PPEE→SE (direct effect)	0.184	0.157	−0.123	0.490	1.173	0.243	–
	PPEE→PTS→SE	0.046	0.055	−0.049	0.179	0.835	0.404	13.7%
	PPEE→ASE→SE	0.230	0.065	0.046	0.305	3.511	0.000	68.7%
	PPEE→PTS→ASE→SE	0.059	0.025	0.009	0.121	2.361	0.018	17.6%
	PPEE→SE (total effect)	0.519	0.138	0.249	0.788	3.77	0.000	–
Moderate perception group	PPEE→SE (direct effect)	0.339	0.058	0.226	0.453	5.856	0.000	–
	PPEE→PTS→SE	0.128	0.019	0.051	0.125	6.789	0.000	72.3%
	PPEE→ASE→SE	0.035	0.012	0.003	0.052	2.871	0.004	19.8%
	PPEE→PTS→ASE→SE	0.014	0.005	0.001	0.020	2.939	0.003	7.9%
	PPEE→SE (total effect)	0.517	0.056	0.408	0.626	9.276	0.000	–
High perception group	PPEE→SE (direct effect)	0.451	0.077	0.300	0.603	5.843	0.000	–
	PPEE→PTS→SE	0.139	0.024	0.046	0.143	5.696	0.000	64.1%
	PPEE→ASE→SE	0.042	0.014	0.006	0.065	2.874	0.004	19.4%
	PPEE→PTS→ASE→SE	0.036	0.010	0.007	0.046	3.609	0.000	16.6%
	PPEE→SE (total effect)	0.668	0.074	0.522	0.814	8.966	0.000	–

PPEE, perceived physical education environment; PTS, perceived teacher support; ASE, academic self-efficacy; SE, student engagement.

## 4 Discussion

Guided by ecological systems theory, social support theory, and social cognitive theory, this cross-sectional study used latent profile analysis to classify undergraduates according to their perceived PE environment, and to examine how different perception levels were related to engagement in PE classes and to the potential mediating roles of perceived teacher support and academic self-efficacy. The results indicated that perceived PE environment was positively associated with student engagement across the low-, moderate-, and high-perception groups. In the mediation analyses, with the exception of the low-perception group—where perceived teacher support did not show a significant independent indirect association between perceived PE environment and engagement—both perceived teacher support and academic self-efficacy exhibited significant individual indirect pathways and jointly formed a serial indirect pathway in the other groups. Taken together, these patterns offer theoretical guidance for efforts to optimize university PE environments, support teacher development, and strengthen resource allocation and institutional incentives.

### 4.1 Association between undergraduates’ perceived physical education environment and student engagement in physical education classes

The present findings indicate that undergraduates' perceived PE environment are positively associated with student classroom engagement, and this pattern is observed across groups with differing levels of perceived environment. This pattern is consistent with ecological systems theory, which posits that individual behavior and development are shaped by multiple facets of the surrounding environment (8). In the context of PE, the four dimensions of the perceived PE environment—curricular design, teacher guidance, institutional environment and material environment—may each be related to student engagement. Empirical work suggests that well-designed PE curricula and adequate facilities can satisfy students' needs for autonomy and competence in physical activity, stimulate enthusiasm for PE courses and strengthen willingness to participate in class (42). A sound institutional environment can provide a fair and orderly learning context, safeguarding students’ rights and opportunities for participation (43). In parallel, extensive prior research indicates that teacher guidance is crucial for fostering students' motivation to engage in classroom learning. In PE classes, such guidance helps students better understand and master PE-related knowledge and skills, sparks interest in exercise and is associated with higher engagement (5). Overall, the present results suggest that undergraduates' perceived PE environment are an important correlate of student engagement. Universities may therefore consider prioritizing the construction and optimization of the PE environment—across curricular design, teacher guidance, institutional arrangements and material resources—as a potential avenue for enhancing students' perceptions and, in turn, supporting their engagement in PE classes.

### 4.2 Mediating role of perceived teacher support between perceived physical education environment and student engagement

In this study, mediation analyses indicated that, in the total sample and among undergraduates with moderate and high perceived PE environment, perceived teacher support accounted for a significant portion of the association between perceived PE environment and student engagement; notably, the relative contribution of this indirect pathway was highest in the moderate-perception group (72.3%). By contrast, the indirect effect of perceived teacher support was not significant in the low-perception group. These results are consistent with social support theory, which proposes that emotional support, informational support and tangible assistance are positively related to well-being and participation. Prior research likewise suggests that higher perceived teacher support is associated with greater interest and engagement in the classroom (5). Considering the three dimensions of perceived teacher support, learning support may facilitate students' understanding and mastery of course objectives; emotional support may enhance affective investment in the course, alleviate stress and anxiety and—through frequent encouragement—be linked to higher levels of classroom engagement; and competence support may strengthen students' self-efficacy. Accordingly, perceived teacher support can reasonably be viewed as a resource that bolsters students' confidence and motivation, conveys teachers' attention and encouragement and, in turn, is associated with greater willingness to participate actively in PE classes. In addition, a more favorable PE environment can provide teachers with greater support and resources, potentially enabling more effective instruction and enhancing students' perceptions of teacher support (44). It is noteworthy that the mediating role of perceived teacher support differed across perception groups. In the low-perception group, the independent indirect effect of perceived teacher support was not significant and emerged only as part of the serial pathway, whereas in the moderate- and high-perception groups, the independent indirect effect of perceived teacher support was significant. One possible explanation is that limitations of the school PE environment—such as insufficient sports equipment, a limited number of PE courses and a lack of emphasis on PE—may restrict the frequency and quality of support that PE teachers can provide (45), resulting in levels of perceived teacher support among students with low perceived PE environment that are too low to show a clear mediating role. Another possibility is that students with low perceived PE environment may hold relatively low expectations and trust toward the broader PE system; in such cases, teacher support may be linked to PE participation only when it simultaneously strengthens students' beliefs in their own competence (i.e., via academic self-efficacy), rather than directly corresponding to more active participation. Therefore, universities should prioritize the construction of the on-campus PE environment and provide teachers with better instructional conditions and resources, to enhance students' perceptions of teacher support and, in turn, improve student engagement in PE classes.

### 4.3 Mediating role of academic self-efficacy in the relationship between the perceived physical education environment and student engagement

In this study, mediation analyses indicated that academic self-efficacy accounted for an indirect association between the perceived PE environment and student engagement in the full sample and across all subgroups, a pattern consistent with social cognitive theory. Students with higher academic self-efficacy tend to be more confident about their performance in PE classes, believe they can successfully complete learning tasks and may therefore be more willing to participate actively in physical activities (34). Prior work also suggests that academic self-efficacy is linked to learning strategies and behaviors, prompting students to seek resources and assistance proactively and to engage in classroom interaction and discussion, thereby supporting better learning outcomes and higher engagement (46). In the present data, perceived PE environment was positively associated with academic self-efficacy. One plausible explanation is that a more favorable PE environment may provide abundant resources and conditions for exercise, ensure sufficient and equitable opportunities for participation and help students adapt to the campus setting, thereby enhancing academic self-efficacy (34), which, in turn, may be associated with higher engagement in PE classes. Notably, the indirect contribution of academic self-efficacy was greatest in the low-perception group (68.7%), whereas in the moderate- and high-perception groups it accounted for less than 20%. One interpretation is that, for students with relatively low perceived PE environment, internal psychological resources such as self-efficacy may play a compensatory or protective role. When external environmental support is lacking or insufficient, students' self-efficacy may become a key correlate of whether they persist in and engage with PE classes (9). In addition, in low perceived PE environment, students may encounter more obstacles, such as crowded facilities, fewer practice opportunities and more rigid institutional arrangements. Under such conditions, only those students who believe they can overcome these constraints may be willing to invest effort and participate actively in PE class activities, which could amplify the mediating role of self-efficacy. By contrast, in the moderate- and high-perception groups, the external environment appears more supportive and opportunities for PE participation are more readily available; environmental features and teacher support may therefore be more directly related to participation, reducing the relative contribution of self-efficacy. Accordingly, universities should optimize PE curricula to increase interest and practicality, strengthen teacher guidance and material conditions, and provide robust learning support to enhance students' academic self-efficacy and, in turn, their engagement.

### 4.4 Serial mediation of perceived teacher support and academic self-efficacy between perceived physical education environment and student engagement

In this study, serial mediation analyses indicated that perceived teacher support and academic self-efficacy together formed an indirect pathway linking perceived PE environment with student engagement, consistent with partial mediation in the moderate- and high-perception groups. In the low-perception group, the association between perceived PE environment and student engagement was fully accounted for by perceived teacher support and academic self-efficacy, underscoring the central role of these factors in that subgroup. This serial pathway illustrates how environmental perceptions may be connected to behavior through social and cognitive psychological processes, integrating perspectives from social support theory and social cognitive theory. Prior research has shown that students with higher perceived teacher support typically report higher academic self-efficacy and that perceived teacher support may be related to student classroom engagement via self-efficacy (35); however, to our knowledge, no studies have systematically examined how perceived PE environment are linked with these variables. The present findings are consistent with a pattern in which more favourable perceived PE environment are associated with higher perceived teacher support, which in turn is associated with higher academic self-efficacy and, ultimately, with higher student engagement in PE classes. The study further showed that, in the low-perception group, the serial mediation of perceived teacher support and academic self-efficacy accounted for the largest proportion of the total indirect effect (17.6%), and that only the academic self-efficacy pathway and the serial pathway were statistically significant. This pattern suggests that, for students with low perceived PE environment, environmental perceptions alone may have a limited association with engagement in PE classes, and perceived teacher support alone may also be insufficiently related to engagement; rather, engagement appears most strongly associated with the combination of teacher support and students' academic self-efficacy. When on-campus PE resources are constrained, it may therefore be particularly important to foster students' academic self-efficacy and to augment teachers' emotional, instructional and competence support as potential strategies to support higher levels of student engagement.

### 4.5 Strengths and limitations

This study has several strengths. First, drawing on multiple theoretical frameworks—ecological systems theory, social support theory and social cognitive theory—it proposed an integrated model that characterizes potential mechanisms linking undergraduates' perceived PE environment with their engagement in PE classes. Second, the use of latent profile analysis identified subgroups with differing levels of environmental perception, which may inform more differentiated intervention strategies. Third, the relatively large sample, spanning multiple regions of China, provides a realistic portrait of Chinese undergraduates and offers a useful reference for university PE reform. Nonetheless, several limitations should be noted. First, the cross-sectional design does not permit causal inferences regarding the relationships among variables. Structural equation modelling and mediation analysis can only indicate the direction and strength of observed associations; without a longitudinal design, firm conclusions about causality cannot be drawn. Future research is therefore encouraged to employ longitudinal designs to track changes over time in perceived PE environment, perceived teacher support, academic self-efficacy and student engagement, thereby probing potential mechanisms more deeply. Second, all data were collected via questionnaires and relied on self-report; although reliability and validity were examined, measurement error may persist. Subsequent studies could further refine the instruments to enhance accuracy and reliability. Third, although the present analyses suggested a pattern consistent with a positive association from perceived teacher support to student classroom engagement, recent work indicates that teachers' perceptions of class-level engagement may, in turn, shape the extent of instructional support they provide. Future studies could therefore adopt cross-lagged or observational designs to examine possible reciprocal relations between these variables. Finally, the sample consisted solely of Chinese undergraduates, which may limit the generalizability of the findings. Future work could broaden the scope to include students from different countries and cultural backgrounds to test the robustness and generality of the conclusions.

## 5 Conclusion

Drawing on ecological systems theory, social support theory and social cognitive theory, this study examined associations between undergraduates' perceived PE environment and their engagement in PE classes, as well as the mediating roles of perceived teacher support and academic self-efficacy. The findings suggest that more favourable perceived PE environment are positively associated with higher student engagement, and that perceived teacher support and academic self-efficacy operate as both individual and sequential mediators in these associations. These results provide theoretical grounding and tentative practical guidance for reform in university PE. Universities may consider optimising the PE environment, strengthening teachers’ support for students and enhancing students' academic self-efficacy as potential strategies to promote active participation in PE classes and to improve instructional quality. Future research could further clarify potential causal relationships among these variables, broaden the scope of investigation and refine measurement tools to deepen inquiry in this field.

## Data Availability

All data supporting the findings of this study are available from the corresponding author upon reasonable request. Requests to access these datasets should be directed to Huizhuo Wu 13507712486@163.com.
